# CD44/CD24 and aldehyde dehydrogenase 1 in estrogen receptor-positive early breast cancer treated with tamoxifen: CD24 positivity is a poor prognosticator

**DOI:** 10.18632/oncotarget.23519

**Published:** 2017-12-21

**Authors:** Yong Wha Moon, Hee-Jung An, Ja Seung Koo, Gun Min Kim, Hyunju Han, Seho Park, Seung Il Kim, Hyung Seok Park, Sewha Kim, Seung Ki Kim, Seung Ah Lee, Sohyun Hwang, Gun Woo Son, Joohyuk Sohn

**Affiliations:** ^1^ Medical Oncology, Department of Internal Medicine, CHA Bundang Medical Center, CHA University, Seongnam, Korea; ^2^ Department of Pathology, CHA Bundang Medical Center, CHA University, Seongnam, Korea; ^3^ Department of Pathology, Yonsei University College of Medicine, Seoul, Korea; ^4^ Division of Medical Oncology, Department of Internal Medicine, Yonsei University College of Medicine, Seoul, Korea; ^5^ Yonsei Cancer Research Institute, Yonsei University College of Medicine, Seoul, Korea; ^6^ Department of Surgery, Yonsei University College of Medicine, Seoul, Korea; ^7^ Department of Surgery, CHA Bundang Medical Center, CHA University, Seongnam, Korea

**Keywords:** breast cancer, estrogen-receptor positive, tamoxifen, resistance, CD24

## Abstract

CD44^+^/CD24^-^ or aldehyde dehydrogenase 1 (ALDH1) has been suggested as a potential marker for breast cancer stem cells. In the cohort of 819 patients with resected ER-positive breast cancer, the ‘5-year relapse group’ within 5 years postsurgery during adjuvant tamoxifen treatment and the ‘non-relapse group’ longer than 9 years postsurgery were defined. Paraffin-embedded tumor tissues were available in 31 patients from 5-year relapse group and 68 from the non-relapse group. CD44/ CD24 and ALDH1 expression was evaluated by immunohistochemical staining. Phenotypes of CD44/CD24 were CD44^+^/CD24^-^ in one patient (1%), CD44^+^/CD24^+^in one patient (1%), CD44^-^/CD24^+^ in 12 patients (12%), and CD44^-^/CD24^-^ in 67 patients (68%). Four patients (4%) showed ALDH1-positivity. Due to the rarity of CD44-positivity or ALDH1-positivity, we dichotomized the patients into CD24-positive status (13%, 13/99 patients) and CD24-negative status (87%, 86/99 patients) only based on CD24 status, and only the status of CD24 was further analyzed. CD24-positivity was higher in the 5-year relapse group (32%) than in the non-relapse group (4%). CD24-positivity was associated with negative PR (P=0.026), higher N stage (P=0.029), and higher histologic grade (P=0.034). However, in the multivariate logistic regression adjusted for the known prognostic factors, CD24-positivity was still a significant predictive factor for 5-year relapse (hazard ratio=8.5; P=0.006). Our results indicated that the expression of CD24 was a significant poor prognostic factor in ER-positive early breast cancer treated with adjuvant tamoxifen. CD24 is worth further investigation as a novel biomarker for tamoxifen resistance beyond general aggressiveness of cancer cells.

## INTRODUCTION

Breast cancer is the most common cancer in women worldwide. Despite advances in detection and development of new treatment approaches, this disease has a high mortality rate due to the emergence of therapy-resistant cancer cells. [1] This resistance phenomenon also applies to tamoxifen therapy. Although in estrogen receptor (ER)-positive breast cancer, adjuvant tamoxifen reduces the risk of recurrence by approximately 50%, [2] many patients who receive tamoxifen as an adjuvant therapy eventually experience disease recurrence. This primary or acquired resistance to tamoxifen is a serious clinical problem. There are at least two major explanations for these observations. One explanation is that all cancer cells acquire resistance, resulting in decreased overall sensitivity to therapy over time. The other explanation is that cells with tumorigenic potential are intrinsically resistant to therapy. In this case, the relative proportion of cells in residual tumors with tumorigenic properties would be expected to increase after treatment. This concept parallels with the cancer stem cell theory.

The cancer stem cell hypothesis holds that since this subpopulation of cells is exclusively able to form tumors, they underpin both disease recurrence and metastasis. [3] Therefore, cancer stem cells have potential major clinical significance. In subsequent studies, CD44^+^/CD24^-/low^ [4] and aldehyde dehydrogenase (ALDH) 1 [5] were suggested as potential candidates for breast cancer stem cell markers. Recently, Li and colleagues reported that tumorigenic breast cancer cells expressing CD44^+^/CD24^-/low^ have primary resistance to conventional chemotherapy and accordingly their proportion increases after chemotherapy. [6] As suggested by linkage between chemotherapy resistance and cancer stem cells, the associations between tamoxifen resistance and breast cancer stem cells can be proposed. Furthermore, although CD44^+^/CD24^-/low^ and ALDH1 were suggested as potential markers for breast cancer stem cells, their clinicopathologic and prognostic significance remains controversial. [5, 7, 8]

The current study aimed to investigate the association between tamoxifen resistance and breast cancer stem cells and clinical implications of CD44/CD24 and ALDH1, potential markers for breast cancer stem cell. Therefore, we evaluated the expression patterns of CD44/CD24 and ALDH1 in ER-positive breast cancer patients receiving adjuvant tamoxifen.

## RESULTS

### Comparison of baseline clinical characteristics between the 5-year relapse versus non- relapse groups

As summarized in Table 1, all thirty-one patints from 5-year relapse group and 68 from the non-relapse group were female, and they all had invasive ductal carcinomas. Between the 5-year relapse group and the non-relapse group, there were no differences in median age, menopausal status, progesterone receptor (PR) and HER2 status, and T stage. However, the 5-year relapse group, showed more advanced N stage (P=0.001) and higher histologic grade (P=0.039) than the non-relapse group. The median time to relapse in the 5-year relapse group was 37.8 months (range, 11.3-65.9 months), and the median follow-up duration in the non-relapse group was 121.4 months (range, 109.3-155.1 months).

**Table 1:** Comparison of patient characteristics between the 5-year relapse versus non-relapse groups

	5-year relapse (n=31)		Non-relapse (n=68)		*P*- value
	n	(%)	n	(%)	
Age					
≤35 years	5	(16.1)	5	(7.4)	0.179
>35 years	26	(83.9)	63	(92.6)	
Menopause					
Pre-	21	(67.7)	45	(66.2)	0.878
Post-	10	(32.3)	23	(33.8)	
PR					
Negative	8	(25.8)	14	(20.6)	0.562
Positive	23	(74.2)	54	(79.4)	
HER2					
Negative	21	(67.7)	50	(73.5)	0.553
Positive	10	(32.3)	18	(26.5)	
T stage					
T1	12	(38.7)	29	(42.6)	0.712
T2-T3	19	(61.3)	39	(57.4)	
N stage					
N0	8	(25.8)	41	(60.3)	0.001
N1	9	(29.0)	17	(25.0)	
N2-N3	14	(45.2)	10	(14.7)	
Histologic grade					
I	3	(9.7)	20	(30.8)	0.039
II-III	28	(90.3)	45	(69.2)	

Abbreviation: PR, progesterone receptor.

### Comparison of IHC results between the 5-year relapse versus non- relapse groups

**Figure 1:**
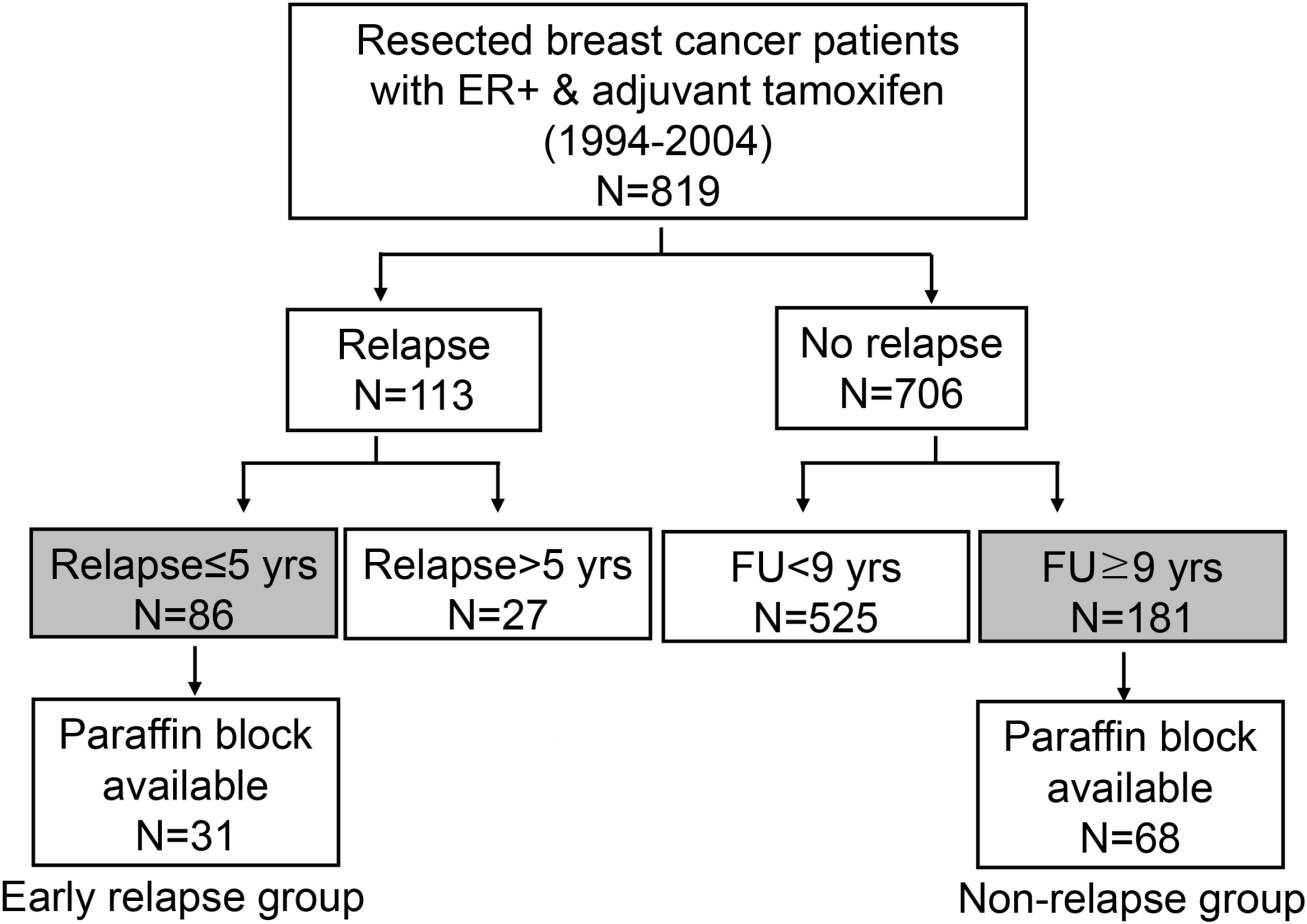
The typical staining patterns for each stem cell marker reflect their subcellular location ALDH1, a cytosolic enzyme, shows cytoplasmic staining (brown). On the other hand, CD44 and CD24 are transmembrane glycoproteins which show membrano-cytoplasmic staining (CD44 in red color, CD24 in brown color).

Typical positive staining patterns for stem cell markers are demonstrated in Figure 1. Phenotypes of CD44/CD24 were CD44^+^/CD24^-^ in one patient (1%), CD44^+^/CD24^+^in one patient (1%), CD44^-^/CD24^+^ in 12 patients (12%), and CD44^-^/CD24^-^ in 67 patients (68%). Four patients (4%) showed ALDH1-positivity. The representative IHC micrographs of ALDH1, CD44 and CD24 are shown in Figure 1. As shown in Table 2, due to the rarity of CD44-positivity (2.9%) or ALDH1-positivity (4.0%), we dichotomized the patients into CD24-positive status (13.1%, 13/99 patients) and CD24-negative status (86.9%, 86/99 patients), only based on CD24 status. Therefore only the status of CD24 was further analyzed to evaluate predictive factors for 5-year relapse. CD24-positivity was higher in the 5-year relapse group (32.3%) than in the non-relapse group (4.4%; P<0.001).

**Table 2:** Comparison of IHC results between the 5-year relapse versus non- relapse groups

	5-year relapse (n=31)		Non-relapse (n=68)		*P*- Value
	n	(%)	n	(%)	
CD24					
Negative	21	(67.7)	65	(95.6)	<0.001
Positive	10	(32.3)	3	(4.4)	
CD44					
Negative	31	(100)	66	(97.1)	1.0
Positive	0	(0)	2	(2.9)	
ALDH1					
Negative	28	(90.3)	67	(98.5)	0.090
Positive	3	(9.7)	1	(1.5)	

Abbreviation: PR, progesterone receptor.

### Analysis of prognostic factors of 5-year relapse

To evaluate the independent prognostic factors of 5-year relapse, significant variables in univariate analysis (P<0.05) including CD24 status were entered into multivariate logistic regression analysis (Table 3). In multivariate analysis, CD24-positivity was still a significant predictive factor for 5-year relapse (hazard ratio=8.5; P=0.006) together with higher N stage. On the other hand, ALDH1- positivity showed a trend for 5-year relapse in univariate analysis (P=0.090).

**Table 3:** Multivariate analysis of predictors of 5-year relapse

	HR(95%CI)	*P*-value
N stage		
N0	1	
N1	3.4 (1.0-11.7)	0.047
N2-N3	5.4(1.6-18.4)	0.008
Histologic grade		
I	1	
II-III	2.0(0.5-8.1)	0.324
CD24		
Negative	1	
Positive	8.5(1.8-39.5)	0.006

Abbreviations: HR, hazard ratio; CI, confidence interval.

^*^Logistic regression.

When the correlation between CD24 positivity and clinicopathological variables was evaluated, CD24-positivity was associated with negative PR (P=0.026), higher N stage (P=0.029), and higher histologic grade (P=0.034) in Table 4.

**Table 4:** Correlation between CD24 positivity and clinicopathological variables

	CD24 negative (n=86)		CD 24 positive (n=13)		*P*- value
	n	(%)	n	(%)	
Age					
≤35 years	9	(10.5)	1	(7.7)	1.0
>35 years	77	(89.5)	12	(92.3)	
Menopause					
Pre-	59	(68.6)	7	(53.8)	0.349
Post-	27	(31.4)	6	(46.2)	
PR					
Negative	16	(18.6)	6	(46.2)	0.026
Positive	70	(81.4)	7	(53.8)	
HER2					
Negative	62	(72.1)	9	(69.2)	1.0
Positive	24	(27.9)	4	(30.8)	
T stage					
T1	37	(43.0)	4	(30.8)	0.549
T2-T3	49	(57.0)	9	(69.2)	
N stage					
N0	44	(51.2)	5	(38.5)	0.029
N1	25	(29.1)	1	(7.7)	
N2-N3	17	(19.8)	7	(53.8)	
Histologic grade					
I	23	(27.7)	0	(0)	0.034
II-III	60	(72.3)	13	(100)	

Abbreviation: PR, progesterone receptor.

## DISCUSSION

The current study originally intended to analyze potential breast cancer stem cell markers in ER-positive early breast cancer. Our result indicating that only very few patients showed potential breast cancer stem cell markers, CD44+/CD24- (one out of 99 patients) or ALDH1 (four out of 99 patients) may be in accordance with previous studies of proof of concept on cancer stem cells. [4, 5] According to the cancer stem cell theory, only a small proportion of tumor cells of less than 5% possess self-renewal capacity and high tumorigenicity. These tumor-initiating cells, called cancer stem cells may be responsible for recurrence and metastasis. Although putative breast cancer stem cells have been suggested to express the CD44+/CD24- marker combination, [4] CD24 alone has been implicated in the regulation of tumor growth and metastasis in previous studies. [9] This prompted us to evaluate if single CD24 expression affects prognosis in adjuvant tamoxifen-treated breast cancer patients, instead of performing further analysis of the relationship between CD44+/CD24- and clinical outcome.

CD24 is a small 27 amino acid glycoprotein at the outer surface of the cell membrane and it is attached to the cell membrane by a glycosylphosphatidyl-inositol (GPI) anchor. Therefore, it is involved in cell adhesion. [10] In breast tissues, previous studies have revealed that CD24 expression was higher in malignant lesions than in benign lesions. [11, 12] Furthermore, CD24 has emerged as a poor prognostic factor in various human cancers [13] including cancers of ovary, endometrium, uterine cervix, stomach, colorectal, esophagus, kidney, bladder, biliary tract, pancreas, non-small cell lung, and prostate as well as breast cancer even though there are some controversies.

In this study, the CD24-positivity rate (13%) was lower than that in previous data (46~85%) from breast cancer studies including more than 100 samples, [14, 15] which may be explained in part by the limitations of IHC assay itself. This means that IHC shows frequently inconsistent results based on the types of antibody, criteria for positivity, and quality of interpretation. However, in this study, CD24 expression was found to significantly correlate with 5-year relapse in ER-positive breast cancer patients during adjuvant tamoxifen treatment, suggesting that CD24 expression may reflect tamoxifen resistance.

At first glance, our data seems to be in contrast with current assertions that the CD44+/CD24-/low subpopulation with tumor-initiating properties is positively correlated with distant metastasis in breast cancer. However, there are several plausible explanations for such a prometastatic role of CD24 expression in human tumor cells. CD24 present on the cell membrane serves as a ligand for P-selectin, and hence, it functions as an adhesion molecule that enhances platelet aggregation. [10, 16, 17] CD24-expressing cancer cells can bind to P-selectin on the endothelial cells and platelets, leading to promotion of extravasation and metastasis of cancer cells. [10, 16, 17] Secondly, several studies have suggested that CD24 also participates in a prometastatic signaling pathway via a regulator such as the chemokine receptor CXCR4. [10, 16, 18] Thirdly, a recent study has revealed that CD24 suppresses the immune response through interaction with Siglec protein expressed on immune cells, [19] contributing to cancer immune escape. However, the exact mechanism by which CD24 results in poor prognosis in various cancers remains unknown and it needs to be further elucidated.

Here, the next question is whether the poor prognostic impact of CD24 is linked to general aggressiveness of cancer cells or specifically of tamoxifen-resistant breast cancer cells. Surowiak et al [20] demonstrated that only adjuvant tamoxifen-treated patients (n=70) showed differential prognosis according to CD24 expression, whereas patients (n=34) without adjuvant tamoxifen did not show differential prognosis. Although the small number of patients without tamoxifen may have led to the lack of difference in prognosis, they found some clue that there may be a correlation between CD24 expression and tamoxifen resistance. Kim et al reported that CD24 expression predicted poor DFS in patients with ER-positive tumors (n=332), and not in patients with ER-negative tumors (n= 312). [21] The current study also demonstrated a similar result as these reports. That is, there was a negative correlation between CD24 expression and prognosis in ER-positive tumors treated with adjuvant tamoxifen. Furthermore, in this study, CD24-positivity more correlated with ER-positive/PR-negative status rather than with both ER-positive and PR-positive status, referring to the luminal B subtype which is known to be relatively resistant to endocrine therapy. In the published microarray data reanalyzed by authors, when MCF7 cells, which are originally ER-positive and tamoxifen-sensitive, acquired resistance after exposure to tamoxifen for several months, tamoxifen-resistant MCF7 cells showed higher CD24 expression compared with tamoxifen-sensitive MCF7 cells, [22, 23] although there is a controversy. [24] Meanwhile, independent microarray analyses of breast cancers showed a consistent inverse correlation between CD24 levels and ER status [25, 26] suggesting that CD24 may be decreased by ER which has transcriptional activity. In addition, *in vitro*, CD24 was reported to be decreased through a direct transcriptional effect depending on ER and histone deacetylases. [27] Accordingly, the authors infer that high expression of CD24 may result from nonsuppression by a lower level of ER and it is potentially associated with tamoxifen resistance among ER-positive tumors. As mentioned above, CD24 has been studied in many cancer types including breast cancer, suggesting a poor prognostic role. In this context, for the originality of our study, we focused on studying potential relationship between CD24 and tamoxifen resistance, on which previous researchers did not give much attention. We found a meaningful novel signal that CD24 may be a tamoxifen-resistance marker. However, it is yet to be clarified whether CD24 directly induces tamoxifen resistance or just a predictive marker of tamoxifen resistance.

Regarding the limitations of the current study, this is a retrospective study, not prospectively designed to investigate tamoxifen resistance. We suppose that there may be a selection bias, because patients at both ends of the prognostic spectrum without a gray zone, which is the ‘5-year relapse group’ during adjuvant tamoxifen treatment and the ‘non-relapse group’ longer than 9 years postsurgery, were selected for this study. However, by comparing these two groups at both ends of the prognostic spectrum, we could identify a novel clue that prognosis of tamoxifen-treated patients is distinguished by CD24 expression, which naturally requires confirmation in larger studies.

In conclusion, in ER-positive early breast cancer, the incidences of CD44^+^/CD24^-^ and ALDH1-positivity by IHC, which are potential breast cancer stem cell markers, were very low. However, the expression of CD24 was a significant poor prognostic factor in ER-positive early breast cancer treated with adjuvant tamoxifen. CD24 is worth further investigation as a novel biomarker for tamoxifen resistance beyond general aggressiveness of cancer cells.

## MATERIALS AND METHODS

### Patients and samples

**Figure 2:**
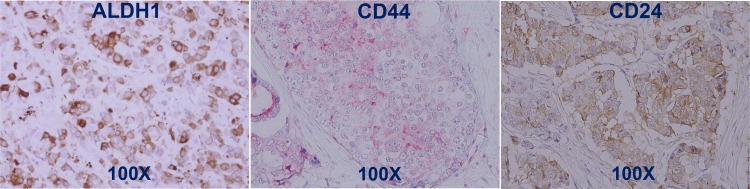
Enrolled patients The ‘5-year relapse group’ within 5 years postsurgery during adjuvant tamoxifen treatment and the ‘non-relapse group’ longer than 9 years postsurgery were included in this study.

Cases were selected from an institutional database containing information on all patients operated for primary breast cancer at Severance Hospital, Yonsei University Health System (Seoul, Korea) from 1994 to 2004. In the cohort of 819 patients with resected ER-positive breast cancer and who received adjuvant tamoxifen therapy, 86 patients relapsed within 5 years postsurgery and 27 patients relapsed after 5 years, whereas 706 patients did not show recurrence at the median follow-up duration of 77.2 months (range, 0.2–187.8 months). Of the 706 non-relapsed patients, 181 patients had a follow-up period longer than 9 years. To assume both ends of the prognostic spectrum, the ‘5-year relapse group’ within 5 years postsurgery during adjuvant tamoxifen treatment and the ‘non-relapse group’ longer than 9 years postsurgery were defined. Paraffin-embedded tumor tissues were available in 31 patients from 5-year relapse group and 68 from the non-relapse group and they were included in the current study (Figure 2). The other paraffin-embedded tumor tissues were damaged during 13~23 years’ storage. Tumor staging was performed according to TNM staging revised in 2002 by the American Joint Committee on Cancer. [28] This study was approved by the Institutional Review Board in Severance Hospital.

### Immunohistochemistry (IHC) and its scoring for CD44, CD24, and ALDH1

Expression of CD44, CD24, and ALDH1 was determined by IHC in formalin-fixed, paraffin-embedded surgical specimens of the 31 patients from 5-year relapse group and 68 from non-relapse group. Tissue microarray (TMA) blocks were generated with punctures in areas which were ensured to have >80% tumor content in each tumor tissue. For IHC, all paraffin sections were cut at 4-μm thickness, deparaffinized through xylene and dehydrated with graded ethanols. Heat induced antigen retrieval pH6 solution was used for antigen retrieval. Endogenous peroxidase activity was blocked by 3% H2O2 in methanol, and primary incubations were performed with mouse monoclonal CD44 antibody (Thermo Scientific) at 1:100 for 60 minutes, mouse monoclonal CD24 antibody (Thermo Scientific) at 1:50, and mouse monoclonal ALDH1 antibody (BD Transduction Laboratories) at 1:100. Subsequently, sections were incubated with DAKO Env+ secondary antibody for 30 min, visualized with 3,3-diaminobenzadine for 10 minutes for chromogenic development, washed and counterstained with hematoxylin. For CD24 and CD44, we used a Envision double-staining system (DAKO).

Positivity was assessed from 0 to 100% of stained cells with cytoplasmic or membranous staining at any intensity by a pathologist who did not know the clinical outcome. CD44 was identified by red color (new fuchsin) and CD24 was identified by brown color [3, 3’-diaminobenzidine (DAB)]. ALDH1 was also identified by brown color (DAB). Positivities for markers were defined as 10% cutoff with membrano-cytoplasmic staining for CD44/CD24 and 5% cutoff with cytoplasmic staining for ALDH1, as previously reported. [21, 29, 30]

### Determination of hormone receptor and human epidermal growth factor receptor 2 (HER2) status

ER (1:100; Thermo Scientific; Fremont, CA) and progesterone receptor (PR; 1:50; Dako, Glostrup, Denmark) status was determined by IHC, where positivity was defined as >1% of the cell population staining positive for the respective receptor. HER2 status was determined by IHC using the HercepTest (1:250; Dako) and scored from 0 to 3+ as previously described in the Herceptin trial. A HER2 IHC score of 3+ was considered positive, whereas cases with a HER2 IHC score of 0 to 1+ were regarded as negative. However, fluorescence *in situ* hybridization test using the PathVysion HER2 DNA Probe Kit (Abbott, IL, USA) was carried out according to the manufacturer's protocol in cases with equivocal (2+) staining, and cases with HER2 gene-to-chromosome 17 ratio >2 were designated as having HER2 overexpression.

### Statistical considerations

Chi-square or Fisher's exact test was used for comparison of categorical variables. Multivariate analyses of predictive factors for 5-year relapse were performed using a logistic regression model. All P-values were two-sided, and the α level was set at 0.05. All statistical calculations were performed using SPSS for Windows, version 17.0 (SPSS, Chicago, IL, USA).
